# Longer and healthier lives for all? Successes and failures of a universal consumer-driven healthcare system, Switzerland, 1990–2014

**DOI:** 10.1007/s00038-019-01290-5

**Published:** 2019-08-31

**Authors:** A. Remund, S. Cullati, S. Sieber, C. Burton-Jeangros, M. Oris, for The Swiss National Cohort, Matthias Egger, Adrian Spoerri, Marcel Zwahlen, Milo Puhan, Matthias Bopp, Martin Röösli, Murielle Bochud, Michel Oris

**Affiliations:** 1 Institute of Demography and Socioeconomics, University of Geneva, Geneva, Switzerland; 2 Swiss NCCR “LIVES - Overcoming Vulnerability: Life Course Perspectives”, University of Geneva, Geneva, Switzerland; 3 Institute of Sociological Research, University of Geneva, Geneva, Switzerland; 4 Department of Readaptation and Geriatrics, University of Geneva, Geneva, Switzerland; 5 Population Research Centre, Faculty of Spatial Sciences, University of Groningen, Landleven 1, 9747AD Groningen, The Netherlands

**Keywords:** Healthy life expectancy, Health inequalities, Healthcare system, Switzerland

## Abstract

**Objectives:**

The ability to translate increases in life expectancy into additional years in good health is a crucial challenge for public health policies. We question the success of these policies in Switzerland, a forerunner of longevity, through the evolution of healthy life expectancy (HLE) across socioeconomic groups.

**Methods:**

Education-specific HLE conditioning on surviving to age 30 was computed for 5-year periods from the Swiss National Cohort, a mortality follow-up of the entire resident population, and the Swiss Health Interview Survey, reporting self-rated health. We compare time trends and decompose them into health, mortality and education components.

**Results:**

Between 1990 and 2015, comparable gains in LE (males: 5.02 years; females: 3.09 years) and HLE (males: 4.52 years; females: 3.09 years) were observed. People with compulsory education, however, experienced morbidity expansion, while those with middle and high education experienced morbidity compression.

**Conclusions:**

Divergence of morbid years by educational levels may reflect unequal access to preventive care due to high out-of-pockets contributions in the healthcare system. This growing gap and the exhaustion of the educational dividend jeopardize future increases in HLE.

**Electronic supplementary material:**

The online version of this article (10.1007/s00038-019-01290-5) contains supplementary material, which is available to authorized users.

## Introduction

In most high-income countries, life expectancy (LE) at birth has continuously increased for the last 150 years (Oeppen and Vaupel 2002), first as a result of reduction in infant and child mortality, and later thanks to gains in survival at older age. These improvements in mortality at old age have raised the question of whether these additional years of life are of good or poor quality.

Three hypotheses were formulated about the relationship between long-term trends in health and mortality in high-income countries. First, some authors predicted that mortality reductions would eventually hit an incompressible level and that healthcare systems would only be able to postpone disease but not death, leading to a compression of morbid years (Fries 1980). This hypothesis was supported by empirical evidence documenting a decline in old age disability (Crimmins 2004; Freedman et al. 2002).

Secondly, others predicted that health care could not escape the “failure of success” consisting in prolonging the life of people with health problems, thus expanding the period lived in poor health (Gruenberg 1977). Empirical evidence supported this hypothesis in the USA, by observing stagnation in the age at onset of health deterioration (Crimmins 2015), and an increase in disease and losses of mobility functioning (Crimmins and Beltrán-Sánchez 2010). Internationally, Global Burden of Disease (GBD) studies concluded that in most countries, between 1990 and 2010, gains in life expectancy were accompanied with a rise in unhealthy years of life (Salomon et al. 2012).

Thirdly, others suggested that gains in mortality are inseparable from improvements in people’s health (Manton 1982). Progresses that push down the risk of death also maintain diseases at less severe stages, thus leading to a dynamic equilibrium between these two dimensions. Empirical evidences for this hypothesis rest on the fact that the expansion of chronic diseases does not translate into increasing prevalence of disability (Hossin et al. 2017; Lin et al. 2016).

The study of these processes across social groups has received little attention. Although it was established that less advantaged subpopulations tend to not only live shorter lives than the average, but also spend a larger part of them in poor health (Majer et al. 2011), we know next to nothing about the evolution of these social differentials over time. In other words, we do not know if compression and expansion of morbidity can coexist in different subpopulations.

In order to tackle this question, we turn to the case of Switzerland. This country has witnessed very rapid gains in life expectancy in the last decades and currently even holds the world record for males. As a forerunner, it can serve as an example of how morbidity levels evolve as new ceilings in longevity are breached. Switzerland’s universal, consumer-directed and tightly regulated healthcare system was praised for its effectiveness and equitable performance (De Pietro et al. 2015), although its reliance on consumers financial contributions poses a risk of reinforcing economic inequalities (Bilger 2008). This makes it a good case study to test whether a consumer-oriented healthcare system can sustain rapid gains in longevity without leaving behind the less privileged share of the population.

## Methods

### Data

Mortality statistics were computed from the Swiss National Cohort (SNC), a longitudinal study of the entire resident population of Switzerland (Bopp et al. 2009). People enter observation during either the 1990 and 2000 “pen-and-paper” censuses, or one of the yearly register-based census since 2010, and leave observation when they appear in a death or emigration register record, or are censored at the end of 2014. In 1990 and 2000, education was known for about 99% of people aged 30 and older (Table 1), but since register-based censuses do not provide education levels, the proportion of cases with missing education in the period 2010–2014 reached about 16% of people aged 30 and older, of which 52% were too young to indicate their education level in 2000, and 48% were new immigrants. To assess the possible biases induced by this limitation, we ran further sensitivity analyses that confirmed the robustness of our findings (Online Resource 2). Highest education was harmonized using the Swiss Federal Statistical Office procedure (Chaze 2005), resulting in three categories: compulsory (primary and lower secondary), upper secondary (e.g. apprenticeship, gymnasium), and tertiary (e.g. universities and universities of applied science). These categories, respectively, match levels 0–2, 3–4, and 5+ of the International Standard Classification of Education 2011 (UNESCO 2011). We extracted age-specific death rates by sex and educational level over 5-year periods. From these rates, we computed life tables and life expectancies conditional on surviving to age 30. In total, we observed 11.65 million individuals over 113 million person-years, and recorded 1.47 million deaths.

**Table 1:** Descriptive characteristics of the dataset (Swiss National Cohort and Swiss Health Interview Survey, Switzerland, 1990–2014)

	Swiss National Cohort (SNC)				
	1990–1994	1995–1999	2000–2004	2005–2009	2010–2014
Person-years (mio)	17.23	22.10	23.52	24.20	26.36
Age (mean)	52.80	53.06	53.36	54.44	54.61
Sex (%)					
Males	47.90	48.11	47.95	48.02	48.52
Females	52.10	51.89	52.05	51.98	51.48
Education (%)					
Compulsory	31.02	27.57	24.42	22.34	19.38
Secondary	49.47	51.72	52.07	53.48	46.57
Tertiary	18.49	19.95	21.86	22.11	18.21
Unknown	1.01	0.75	1.65	2.08	15.84

	Swiss Health Interview Survey (SHIS)				
	1992	1997	2002	2007	2012
Sample size	11,676	10,333	16,835	15,811	17,296
Survey participation rate (%)	70.8	68.8	63.9	66.3	53.1
Suboptimal health, weighted (%)	4.13	4.20	3.90	4.78	5.06

Health statistics were computed from the Swiss Health Interview Survey (SHIS), a cross-sectional survey conducted every 5 years since 1992 by the Swiss Federal Office for Statistics. It is designed to be representative of all residents of Switzerland aged 15 and older, randomly selected following a two-stage stratified sampling strategy. In our study, we included 72,123 people aged 30 or older who participated in any of the five available waves (1992–2012). Respondents who did not provide a valid response to their self-rated health status (*N* = 42) or educational level (*N* = 130) were excluded. The final study population includes 71,951 individuals. Survey weights were used to correct for the survey sampling strategy and non-participation biases. Overall participation rate was 64.6%, with a regular decline over waves (Table 1).

### Methods

We defined healthy life expectancy (HLE) using self-rated health (SRH). SRH is a well-known single item with a satisfactory reliability (Cox et al. 2009) and validity (DeSalvo et al. 2006). In each SHIS wave, participants were asked to rate their general health status on a five-point Likert scale. The phrasing of the question and the response items were, however, not identical in each survey language and wave. We examined the best recoding scheme of the SRH item to maximize its equivalence across time and survey language and found that dichotomizing the best three categories (very good, good, moderate) against the two worst ones (poor, very poor) was the best solution (Online Resource 1).

HLE by sex, educational level and period was computed using the Sullivan method (Sullivan 1971). This widely used measure consists in weighing the person-years lived of a period life table by the prevalence of good health. Additionally, we computed confidence intervals for both LE and HLE using Monte Carlo methods. For each point estimate, we generated 1000 simulated values by drawing random deviates, from a Poisson distribution for age-specific death counts, and from a binomial distribution for good health prevalence. We then extracted the 95% quantiles of these 1000 values, which can be interpreted as confidence intervals in a resampling framework. HLE was not computed for people with missing education, since this category did not appear in the health data.

We measured morbidity expansion/compression by the temporal change in years spent in bad health (YBH), defined as the difference between LE and HLE. From an absolute point of view, YBH increase if morbidity is expanding, decrease if morbidity is compressing, and remain stable in a case of equilibrium. We decompose this change by sex, age and education level along three dimensions: mortality rates, good health prevalence and educational composition, using stepwise replacement techniques (Andreev et al. 2002). Missing education was not included in the health dimension since this category does not appear in the health data.

## Results

Table 1 shows a regular increase in the number of person-years. It reflects the growth of the national population due to immigration and gains in longevity, as shown by the increasing mean age of the sample. The educational composition gradually improved, as younger generations attained a higher education level than their predecessors. The share of people with a low level of education decreased by about 9% between 1990–1994 and 2005–2009, while those with a middle and high level of education increased by 5% and 4%, respectively. The recent apparent reversal in this trend is an artifact of the introduction of register-based censuses (see in “Methods” section).

**Fig. 1:**
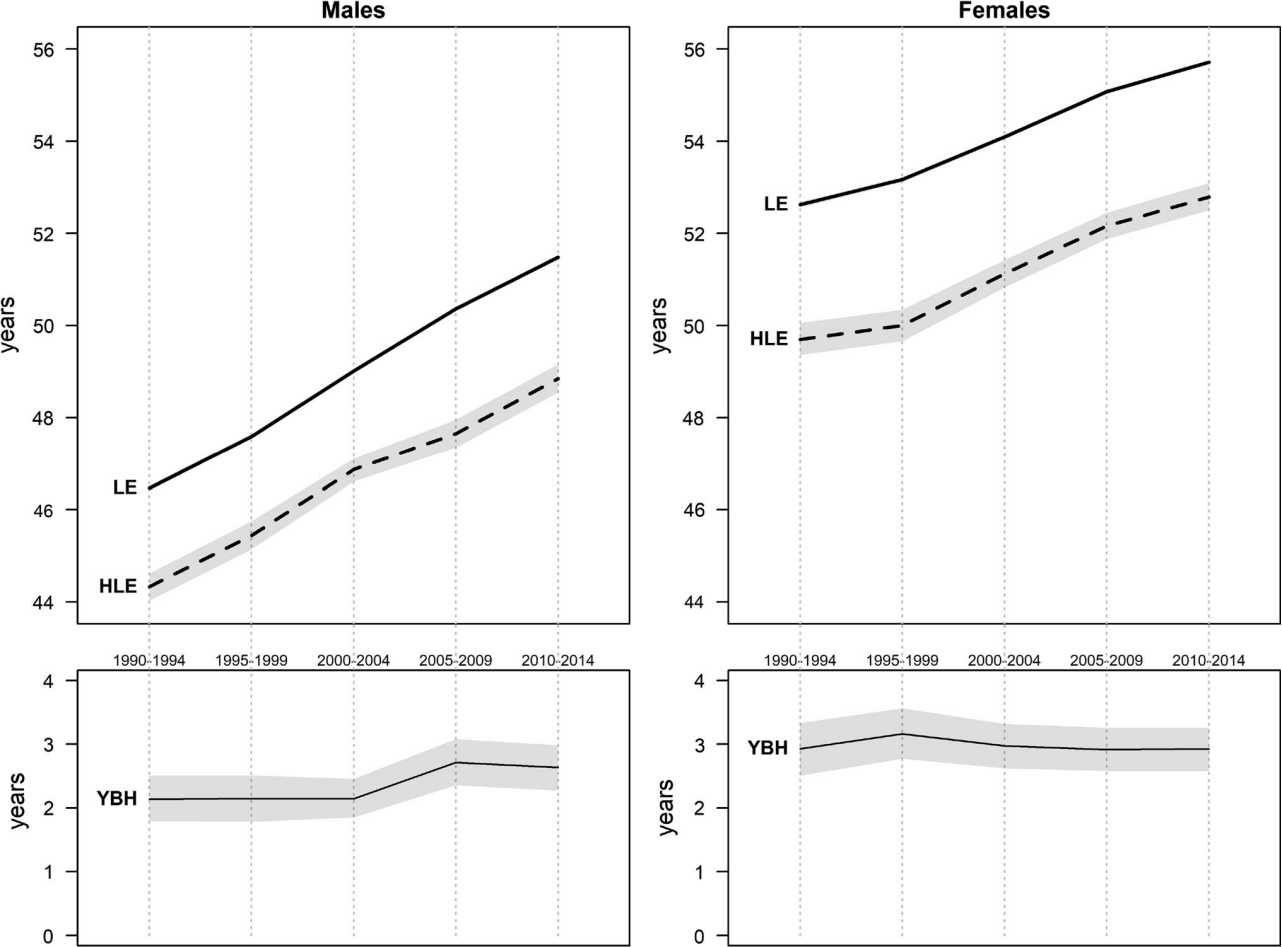
Life expectancy (LE), Healthy life expectancy (HLE), and Years of bad health (YBH) by sex (Swiss National Cohort and Swiss Health Interview Survey, Switzerland, 1990–2014)

Figure 1 confirms the continuous progresses in life expectancy (LE) in Switzerland. At age 30, LE currently reaches 51.5 years for males and 55.7 years for females, meaning that a 30-year-old living in Switzerland can currently expect to reach on average a lifespan of 81.5 years for a man and 85.7 years for a woman. These figures assume, however, that mortality conditions remain stable in the future and do not necessarily reflect the prospects of cohorts.

Meanwhile, healthy life expectancy (HLE) at age 30 currently reaches 48.8 years for males and 52.8 years for females. Males are thus at an absolute disadvantage compared to females, but in relative terms both sexes spend about 95% of their lives in a state of good self-rated health. In terms of years of bad health (YBH), females are even slightly disadvantaged.

Both LE and HLE underwent important changes over the 25 years of observation. LE increased by 5.02 years (± 0.11) for males and 3.09 years (± 0.11) for females. This increase followed a monotonic trend for both sexes, albeit at a different pace. Male LE increased by about 0.25 year per year. Progresses in female LE were slower, varying between 0.1 year per year (in the 1990s and 2010s) and 0.2 year per year (between 1995 and 2009).

Trends in HLE followed a similar pattern. Males enjoyed an increase of 4.52 years (0.23 per year on average), against 3.09 years (0.15 per year) for women. Moreover, periods of fast increase in LE such as 1995–2009 coincide with an acceleration in HLE, and conversely, periods of slower mortality improvement such as the early 1990s and 2010s are also marked by smaller gains in morbidity. The parallel trends in LE and HLE lead to a remarkably stable YBH over the 25 years of observation, hovering at about 2 years for males and 3 years for females. The only exception is the early 2000s for males, which saw an expansion of morbidity by half a year.

**Fig. 2:**
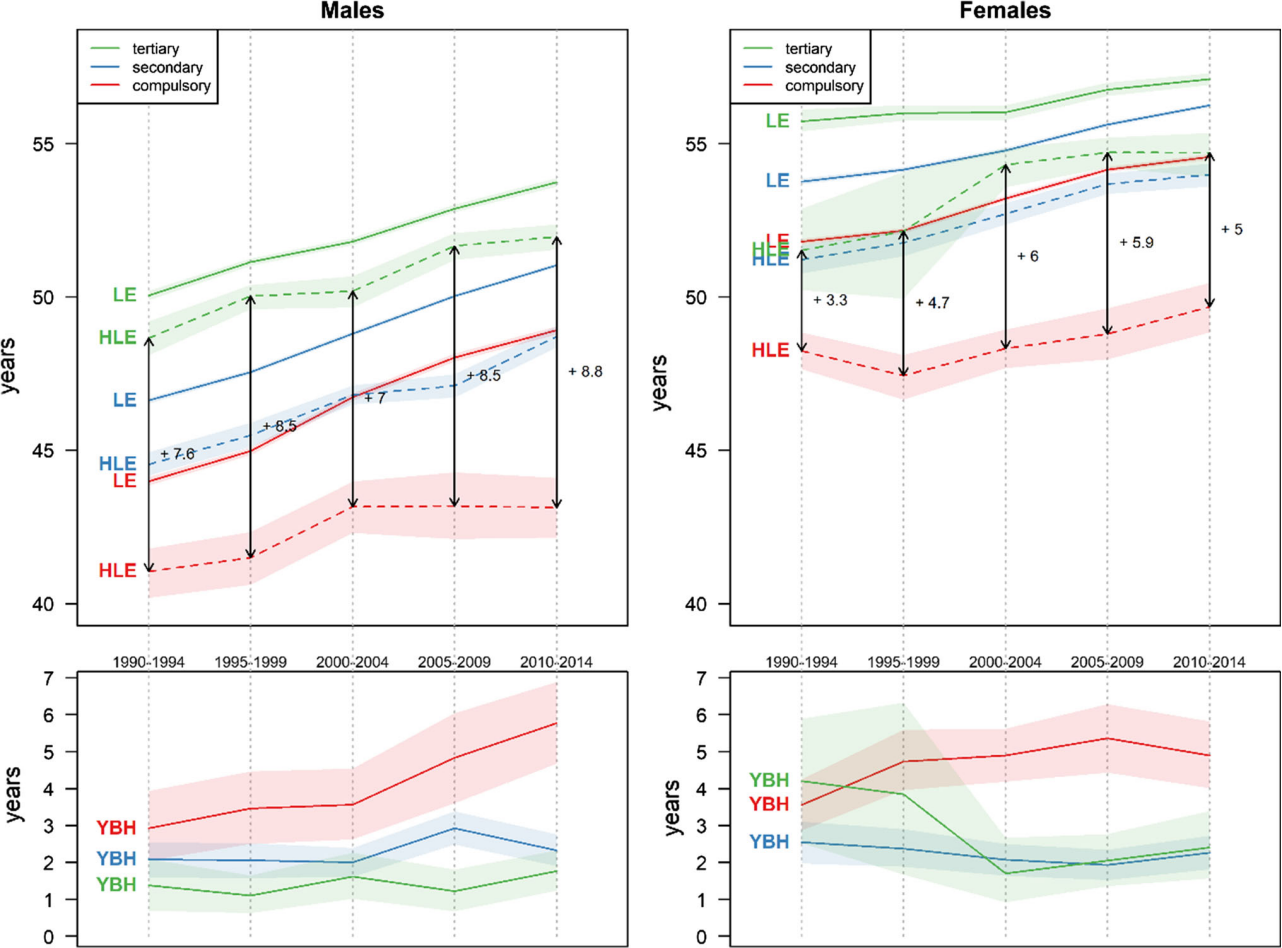
Life expectancy (LE), Healthy life expectancy (HLE), and Years of bad health (YBH) with 95% confidence intervals by education level and sex (Swiss National Cohort and Swiss Health Interview Survey, Switzerland, 1990–2014)

Figure 2 disaggregates these results by educational level. It shows a striking decrease in the educational gradient in LE. In the early 1990s, the gap between people with compulsory and those with tertiary education amounted to about 6 years for males and 4 years for females. Two decades later, these gaps have narrowed to less than 5 years and 2.5 years, respectively. This convergence took place progressively over the whole period, but was especially fast between the late 1990s and early 2000s due to a temporary deceleration of the improvement among people with tertiary education.

On the contrary, the educational gradient in HLE increased between 1990 and 2014. The gap in HLE between people with compulsory and tertiary education increased from 7.6 to 8.8 years among men, and from 3.3 to 5.0 years among women. This process was due to a much slower increase, and for one period even a decrease (women 1995–1999), in HLE for people with compulsory education compared to those with secondary and tertiary education. Among those, the former even tended to catch up with the latter, and for women they often did not differ significantly.

Consequently, while YBH remained stable in the overall population, they clearly increased for people with compulsory education. This process started in the 1990s for women, when YBH expanded from 3.5 to 5 years. For low-educated men, YBH first slightly increased from 3 to 3.5 years in the 1990s and then surged to about 6 years in the last period. Between 1990 and 2014, the educational gradient in YBH went from non-significant to a dichotomization between people with compulsory education on the one hand, and people with secondary and tertiary education on the other hand.

**Fig. 3:**
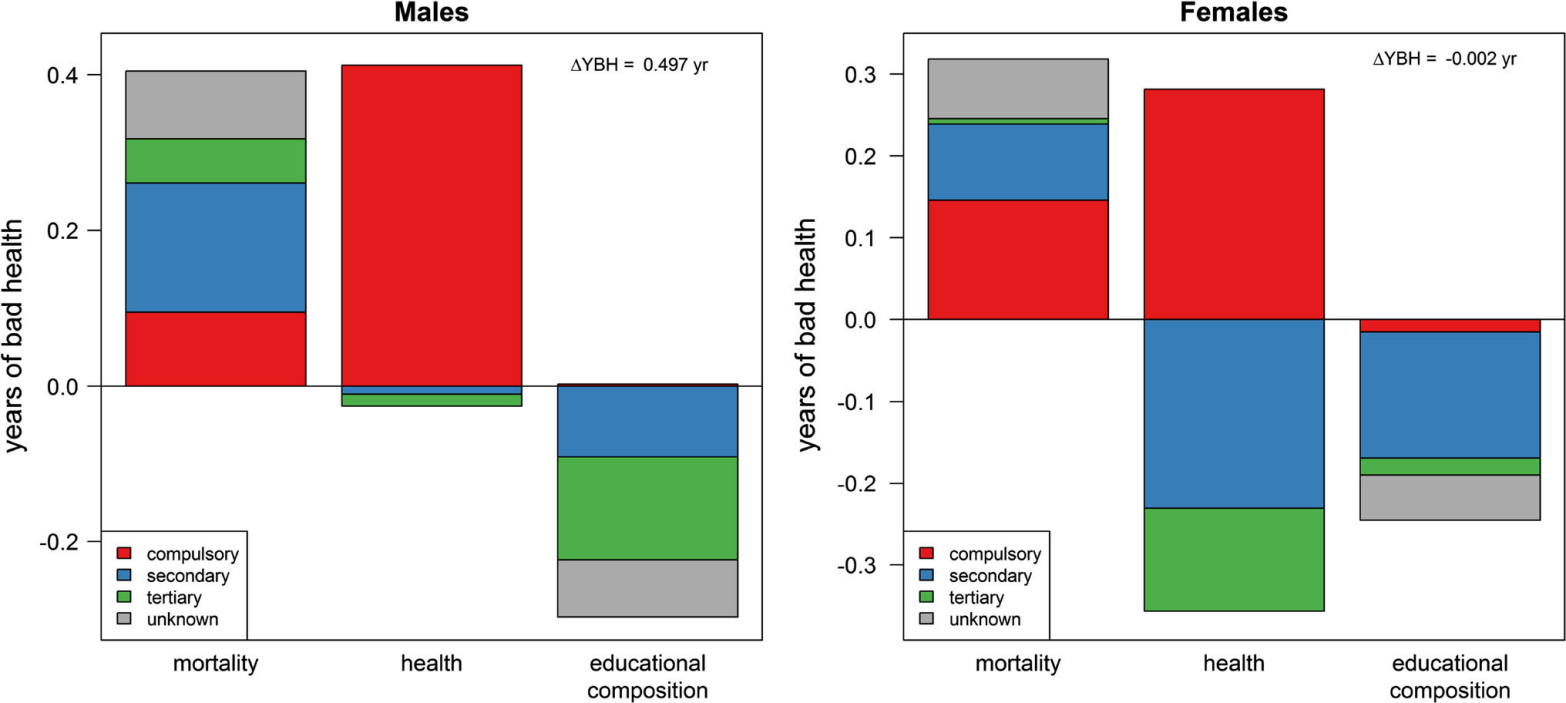
Contributions of mortality, health and educational composition to the change in years of bad health between 1990–1994 and 2010–2014 (Swiss National Cohort and Swiss Health Interview Survey, Switzerland, 1990–2014)

Figure 3 indicates the contributions of mortality, health and educational composition to the change in overall YBH from 1990–1994 to 2010–2014 (ca. + 0.5 for males and 0 for females). Decreasing mortality generated positive contributions that were partly compensated by negative contributions of improving health, but only for women with more than compulsory education. People with compulsory education displayed positive contributions due to deteriorating health, while men with higher education did not experience a change in health. The overall stability was thus possible only thanks to an improvement of the educational composition caused by a reduction in the share of people with low education.

## Discussion

Our analyses show that in the last 25 years Switzerland experienced important gains in LE, similar to the pace of the world’s record (Oeppen and Vaupel 2002). Such gains were equally matched by improvements in self-rated HLE. This resulted in a remarkably stable number of YBH, except for a short increase for males during the early 2000s. The finding that overall mortality and morbidity followed similar trends supports the hypothesis of dynamic equilibrium in the general population (Manton 1982).

In terms of the educational gradient, our results confirm larger-scale studies showing that HLE generally displays a larger gap than LE (Mäki et al. 2013). More unexpectedly, these differentials follow different trends: diverging for HLE and converging for LE. Consequently, people with low and high levels of education experienced morbidity expansion and compression, respectively. This conclusion suggests that the relationship between mortality and morbidity can be more complex than large international studies present it. Rather than a single process, morbidity compression or expansion, each country can experience simultaneously each of these processes across multiple subpopulations.

While many studies document a divergence of LE by various measures of social classes (Bosworth 2018; Brønnum-Hansen and Baadsgaard 2008; Cambois et al. 2001; Deboosere et al. 2009; Hattersley 1999; Sasson 2016), only a handful compare trends of educational differentials in LE with those of HLE (Cambois et al. 2001; Crimmins and Saito 2001; Perenboom et al. 2005; Renard et al. 2019), and only one (Klotz 2010) uses self-rated health. Using Austrian data between 1981 and 2006, it shows a convergence in LE between middle and high levels of education, while people with low levels of education trailed behind. In terms of HLE, it shows a divergence between middle/high and low levels of education, resulting in a sharp increase in YBH for people with low levels of education. The striking similarity of these findings suggests that our results may reflect a wider phenomenon extending beyond Switzerland to other high-income countries.

A key aspect of this process is that, as in the case of Austria, the divergence in HLE is caused by the fact that people in the lowest educational group experienced an expansion of morbidity, while people with secondary education display either a stable (males) or a decreasing (females) gap with their counterparts with tertiary education (Fig. 2). Consequently, only the lowest educational group contributes positively to the change in YBH due to a worsening SRH level (Fig. 3). What our results are showing is thus essentially a process of marginalization of people with compulsory education. This interpretation is supported by the fact that, internationally and in Switzerland, younger birth cohorts with lower levels of education display worse levels of SRH and that the gap with other educational levels is increasing over time (Hu et al. 2016; Volken et al. 2017).

This process is probably reinforced by the fact that this group represents a decreasing share of the total population. Following the democratization of higher education, this group has become smaller and contains a larger proportion of individuals who are vulnerable in other dimensions of their life course (Oris et al. 2017). Eventually, those who stay behind suffer from a disadvantage not only due to their persistent relative position, but also because of their marginalization in the overall population (Bühlmann et al. 2012).

This marginalization is visible on the labor market. Over the last 20 years, the difference in unemployment rates between people with low and middle/tertiary education has strongly increased in Switzerland. In 2001, when overall unemployment was especially low, 2.8% of males and 3.7% of females with compulsory education were unemployed, compared to 1.1% and 3.1% in the total population. In 2014, people with compulsory education endured almost a 10% unemployment rate, against 4% for those with secondary and tertiary education (OFS 2018).

It is hard not to draw a connection between these evolutions in the labor market and the deterioration of health among people with compulsory education, especially in working ages. The worsening health status of people with compulsory education contributed to an increase in YBH of 0.41 years among males, and 0.28 years among females (Fig. 3). Almost all of this contribution came from people between 40 and 60 years of age (Online Resource 3). This age group makes up 93% of the male contribution, while for females it even exceeds the contribution of health all age groups combined, because of negative contributions from younger and older ages.

While these evolutions can explain the divergence in HLE by educational level, they do not explain the concomitant convergence in LE, because mortality and health are usually supposed to be driven by the same factors (Link and Phelan 1995). One would thus expect any change in one of these dimensions, such as we highlighted in the case of unemployment, to have an impact on the educational gradient of both LE and HLE. While growing socioeconomic differentials are likely driving these trends, we argue that their effects might be moderated differently on health and mortality by the specificities of the Swiss healthcare system.

A possible explanation for these opposite trends in terms of LE and HLE differentials is the unequal ability of the Swiss healthcare system to provide curative and preventive medicine to everyone. On the one hand, its universal and decentralized access to health care, wide range of curative and rehabilitative treatments, and high level of health expenses per gross domestic product favor a performant curative medicine for all (De Pietro et al. 2015). These qualities of Switzerland’s public health system explain its place among the world leaders in life expectancy. It also probably explains its ability to force a convergence in terms of mortality risks across educational levels.

On the other hand, out-of-pocket payments for health and unmet health care needs for financial reasons are among the highest in OECD countries (De Pietro et al. 2015). This forces those with compulsory education to forego medical appointments (and thus preventive treatments) for financial reasons twice as much as people with secondary and tertiary education (OFS 2017). These social inequalities in preventive health care could explain why morbidity is expanding among the least advantaged.

Although this hypothesis cannot be proven at this stage, it is supported by a growing amount of circumstantial evidence. Several studies have revealed that, in Switzerland, disadvantaged patients tend to forego medical treatments much more than the average. National surveys showed that, between 2007 and 2014, the percentage of people that forego medical treatment has increased among people with compulsory education, while it remained stable for people with tertiary education (OFS 2017). It was also reported that people with compulsory education are significantly less likely to screen for cervical and prostate skin cancers (Burton-Jeangros et al. 2017; Guessous et al. 2016). Another study discovered that foregoing a medical appointment increased from 22 to 34% among people earning less than CHF 3000 per month between 2007 and 2010 (Guessous et al. 2012). These different studies show that preventive care is provided less and less equally across all socioeconomic groups in Switzerland.

Our results highlight the forces and weaknesses of the Swiss healthcare system, often praised for its ability to efficiently convert heavy financial investments into good public health achievements. They hint that its consumer-directed and tightly regulated aspects enable it to offer high life expectancy to most of its citizens, but its high out-of-pocket expenses are proving an obstacle to the democratization of similar gains in healthy life expectancy. Unless this issue is addressed, it is likely that future improvements in public health will be hampered. This is true for HLE as the improvement in the educational structure of the population that have supported its growth cannot continue forever, but also for LE if we consider that people exposed to bad health suffer from higher mortality risks later in life.

Our study has several limitations that we tried to address (Online Resources). Changes in SRH phrasing in French and Italian could affect conclusions, but sensitivity analyses restricted to German language confirmed the national results. The lack of information on people’s education after 2000 means that a significant share of people had to be treated in a separate unknown category, but sensitivity analyses restricted to people present in 1990 confirm our results. Conclusions on HLE are based on self-reported general health, which does not allow distinguishing between people’s perception of their somatic and mental health.

## Electronic supplementary material

Below is the link to the electronic supplementary material.

Supplementary material 1 (DOCX 923 kb)
