# Supplementary material for: Longer and healthier lives for all? Successes and failures of a universal consumer-driven healthcare system, Switzerland, 1990–2014
Source: Int J Public Health. 2019 Aug 31;64(8):1173–81. doi: 10.1007/s00038-019-01290-5 (PMC6811388; doi:10.1007/s00038-019-01290-5)
Supplement: Supplementary file 1 — Supplementary material 1 (DOCX 923 kb) [file 38_2019_1290_MOESM1_ESM.docx]

# Electronic Supplementary Material

# Article title: Longer and healthier lives for all? Successes and failures of a universal consumer-driven health care system, Switzerland, 1990-2014.

# Journal: International Journal of Public Health

# Online Resource 1: Sensitivity analysis of the changes in wording of SRH

Self-rated health (SRH) has been present under three different forms with the Swiss Health Interview Survey (SHIS), from 1992 to 2012 (Table 1). The first one was used in 1992, 1997 and 2002, the second one in 2007, and the third one in 2012. The changes from one version to another do not affect all languages equally. Regarding the question, the French questionnaire did not mention explicitly health in the first form, contrarily to the other languages (German: *gesundheitlich*, Italian: *salute*). This incoherence was corrected in the second and third versions (French: santé).

In terms of the response items, the five options also varied across languages and versions, with the exception of the German form, which remained identical in all forms. The best two options (*Very good* and *Good*) are totally stable over time and completely comparable across languages. The last two worst categories (*Bad* and *Very bad*) also remain almost identical, with the exception of the first Italian version, which proposed "lighter" formulations (*Not very good* and *Bad*). The largest variation comes from the central item, whose formulation ranges from *Mediocre* to *Relatively good*, taking in total seven different formulations.

|  | **Language** | **Form 1** | **Form 2** | **Form 3** |
| --- | --- | --- | --- | --- |
| ***Question wording*** | German | Zuerst gerade das Nächstliegende: Wie geht es Ihnen zur Zeit gesundheitlich?  *First and foremost: how are you doing at the moment in terms of heath?* | Wie ist Ihre Gesundheit im Allgemeinen?  *How is your health in general?* | Wie ist Ihr Gesundheitszustand im Allgemeinen? Ist er…  *What is your health status in general? Is it…* |
|  | French | Commençons par l'essentiel : Comment allez-vous en ce moment ?  *Let's start with the essential: how are you doing at the moment?* | Comment est votre santé en général ?  *How is your health status in general?* | Comment est votre état de santé en général ? Est-il…  *How is your health status in general? Is it…* |
|  | Italiano | Cominciamo dall'essenziale: Come sta di salute in questo momento?  *Let's start with the essential: how is your health at the moment?* | Come è la Sua salute in generale?  *How is your health in general?* | Come va in generale la sua salute?  *How is your health in general?* |
| ***Response items*** | German | Sehr gut / *Very good*  Gut / *good*  Mittelmässig / *Mediocre*  Schlecht / *Bad*  Sehr schlecht / *Very bad* | Sehr gut / *Very good*  Gut / *good*  Mittelmässig / *Mediocre*  Schlecht / *Bad*  Sehr schlecht / *Very bad* | Sehr gut / *Very good*  Gut / *good*  Mittelmässig / *Mediocre*  Schlecht / *Bad*  Sehr schlecht / *Very bad* |
|  | French | Très bien / *Very good*  Bien / *Good*  Comme ci, comme ça (moyen) / *so, so (moderate -average)*  Mal / *Bad*  Très mal / *Very bad* | Très bonne / *Very good*  Bonne / *Good*  Moyenne / *Moderate-average*  Mauvaise / *Bad*  Très mauvaise / *Very bad* | Très bon / *Very good*  Bon / *Good*  Assez bon / *Relatively good*  Mauvais / *Bad*  Très mauvais / *Very bad* |
|  | Italiano | Molto bene / *Very good*  Bene / *Good*  Mediamente / *Average*  Non molto bene / *Not very good*  Male / *Bad* | Molto buona / *Very good*  Buona / *Good*  Normale / *Normal*  Scadente / *Poor*  Molto scadente / *Very poor* | Molto bene / *Very good*  Bene / *Good*  Discretamente / *Fair*  Male / *Bad*  Molto male / *Very bad* |

Table 1: original wordings on the three forms for self-rated health in the Swiss Health Interview Survey. Form 1 corresponds to waves 1992, 1992 and 2002, form 2 to wave 2007, and form 3 to wave 2012. (English literal translations proposed by the authors in italics)

This thorough analysis of changes in the formulation of both questions and response items to the SRH suggests a clear breach of continuity at the most detailed level of measure. It however also shows that the highest and lowest categories are not affected by this problem in any language. This justifies the use of a dichotomization into "good" and "bad" health, to smooth the transitions between versions of the form. The fact that, for the Italian language, there was no worse category than *Bad* in the first version probably means that it includes a proportion of people who would have otherwise qualified their health as *Very bad*. Since these two categories would anyway be merged in a dichotomization, this does not seem to be a problem. The real question thus is how to treat the middle category, to include it with "good" or "bad" health.

Figure 1 (below) replicates Figure 1 of the article, considering this time the middle category as "bad health". Measured according to this latter definition, trends in HLE display strong and implausible variations over time. Specifically, the drop in HLE recorded in the last period in both sexes is probably due to the fact that the label of the middle category is more positive than in previous versions in French and Italian (from *Normal / Average* to *Relatively good / Fair*). This means that the share of people classified as "bad health" was artificially inflated compared to previous waves.

In conclusion, as middle categories in the second and third form of the Italian and French versions of the questionnaire move closer to "good health", their inclusion in the "bad health" category becomes more questionable. They are probably better conceived as refinements of the *Good* health item from previous questionnaires. We considered thus that including it with "good health" is a more coherent solution.


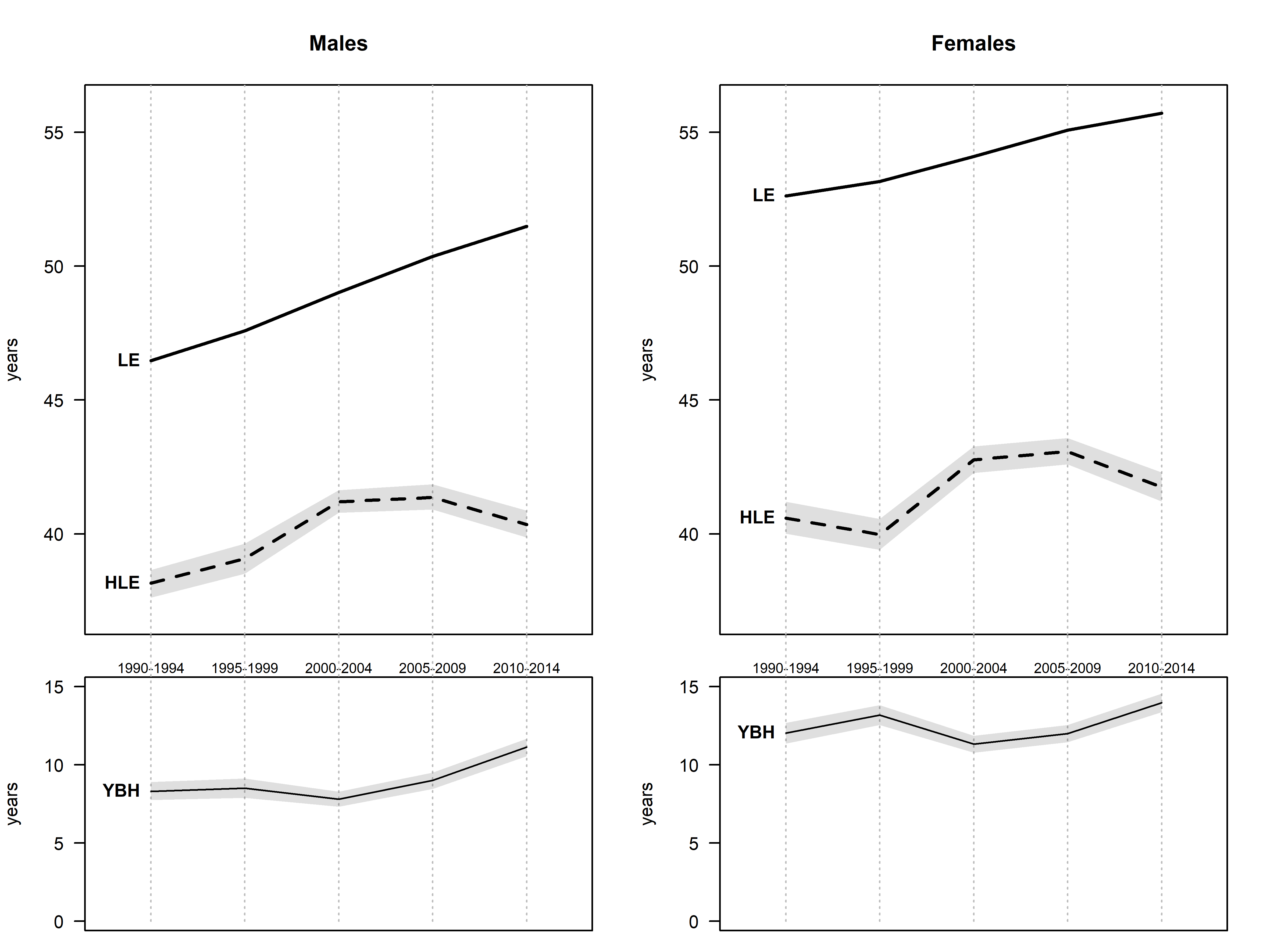


Figure 1: Life expectancy (LE), Healthy life expectancy (HLE), and Years of bad health (YBH) with middle category as "bad health" (Swiss National Cohort and Swiss Health Interview Survey, Switzerland, 1990-2014)

In order to make sure that our solution efficiently solves the problem of changing definitions of SRH, we repeated our analysis (with the middle category as "good health") on a restricted sample of the population living in the German-speaking region. German speakers in Switzerland account for about two thirds of the population, which means that it heavily influences the results obtained on the national population. We should therefore broadly observe the same trends.

The results (Figure 2) are very similar to the ones obtained on the national population. It notably shows a similar increase in YBH for males between 2000-04 and 2005-09 as the one observed in the national population. This confirms that this jump is not an artifact of the changing formulation of SRH, since the German formulation of the responses did not change over time. Small differences can be observed with the results obtained on the national population, which can be due either to stochasticity (see the relatively wide confidence intervals), or to specificities of the German-speaking region compared to the French- and Italian-speaking regions.


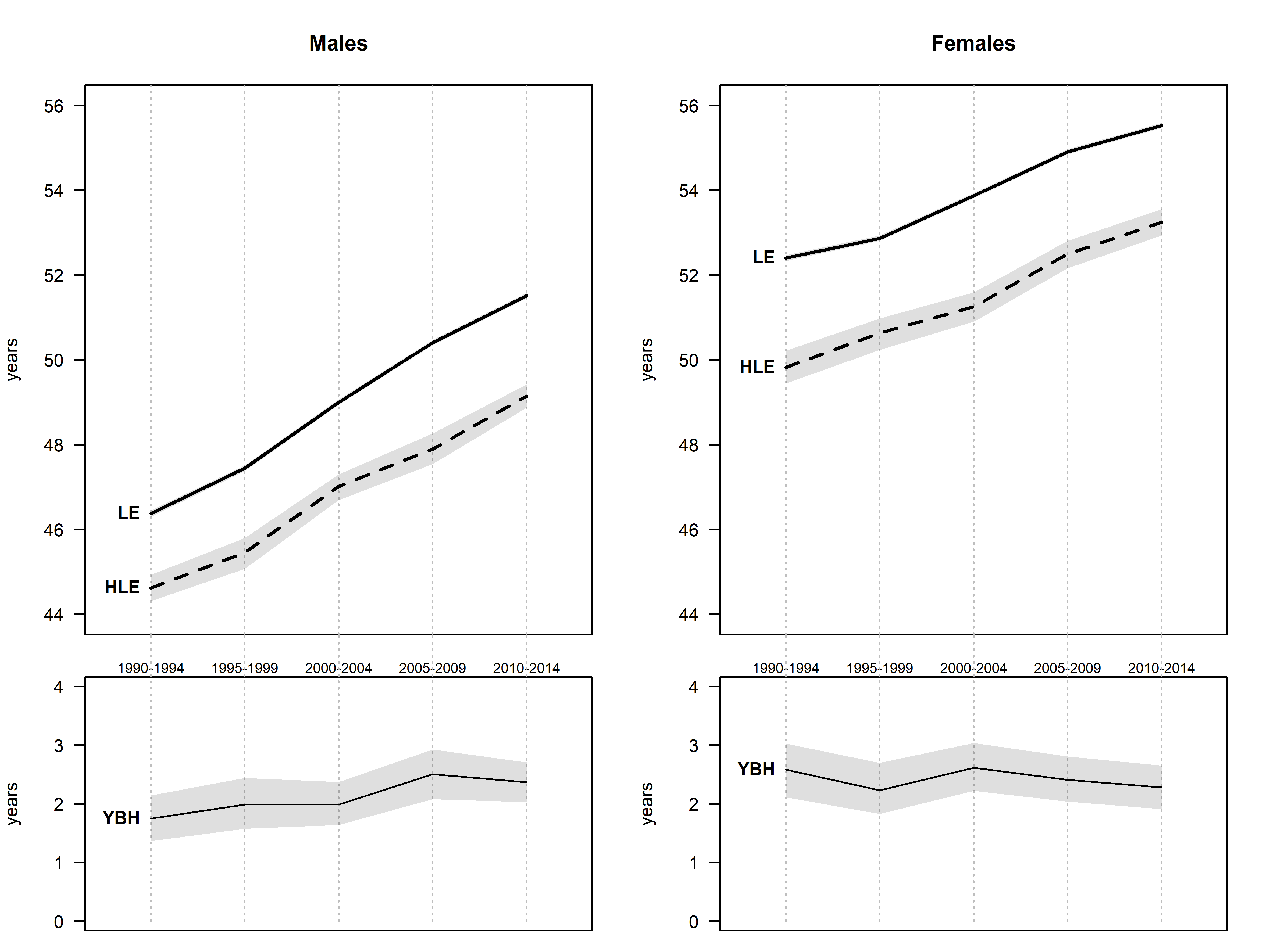


Figure 2: Life expectancy (LE), Healthy life expectancy (HLE), and Years of bad health (YBH) with middle category as "bad health" in the German-speaking population (Swiss National Cohort and Swiss Health Interview Survey, Switzerland, 1990-2014)

# Online resource 2: Sensitivity analysis of missing education and immigration

As mentioned in the paper, a weakness of our data is the lack of information on the educational level of people who were not present either at the 1990 or the 2000 census. This is because the new register-based census inaugurated in 2010 does not contain information on education. Consequently, the proportion of unknown education jumps from 1-2% to 15%. About half of this increase is due to new migrants, while the other half concerns people who were present at the 2000 census but were too young to declare their level of education. This could generate a bias if the educational structure for people who enter observation after 2000 is different than that of the preexisting population.

An argument for the existence of such a potential bias is that, in Switzerland as in many countries, migrants live on average longer (have lower mortality rates) than natives (1). It is thus possible that, by excluding this newly arrived population from the education-specific analyses, life expectancies are underestimated in the last period. Furthermore, this bias may be stronger for highly educated people since, especially since Switzerland entered the Schengen Area, migrants have on average a higher education than natives in Switzerland (2).

In order to exclude these potential biases, we restricted our sample to people who were present at the 1990 census. Treating our data as a cohort eliminates the healthy migrant effect. Both overall (Figure 3) and education-specific (Figure 4) results are very close to the ones obtained in the article using the current population. In particular, YBH keeps its stable pattern, with an increase for males between 2000-04 and 2005-09, and education-specific results fall within 0.1 year of life expectancy of the original figures.


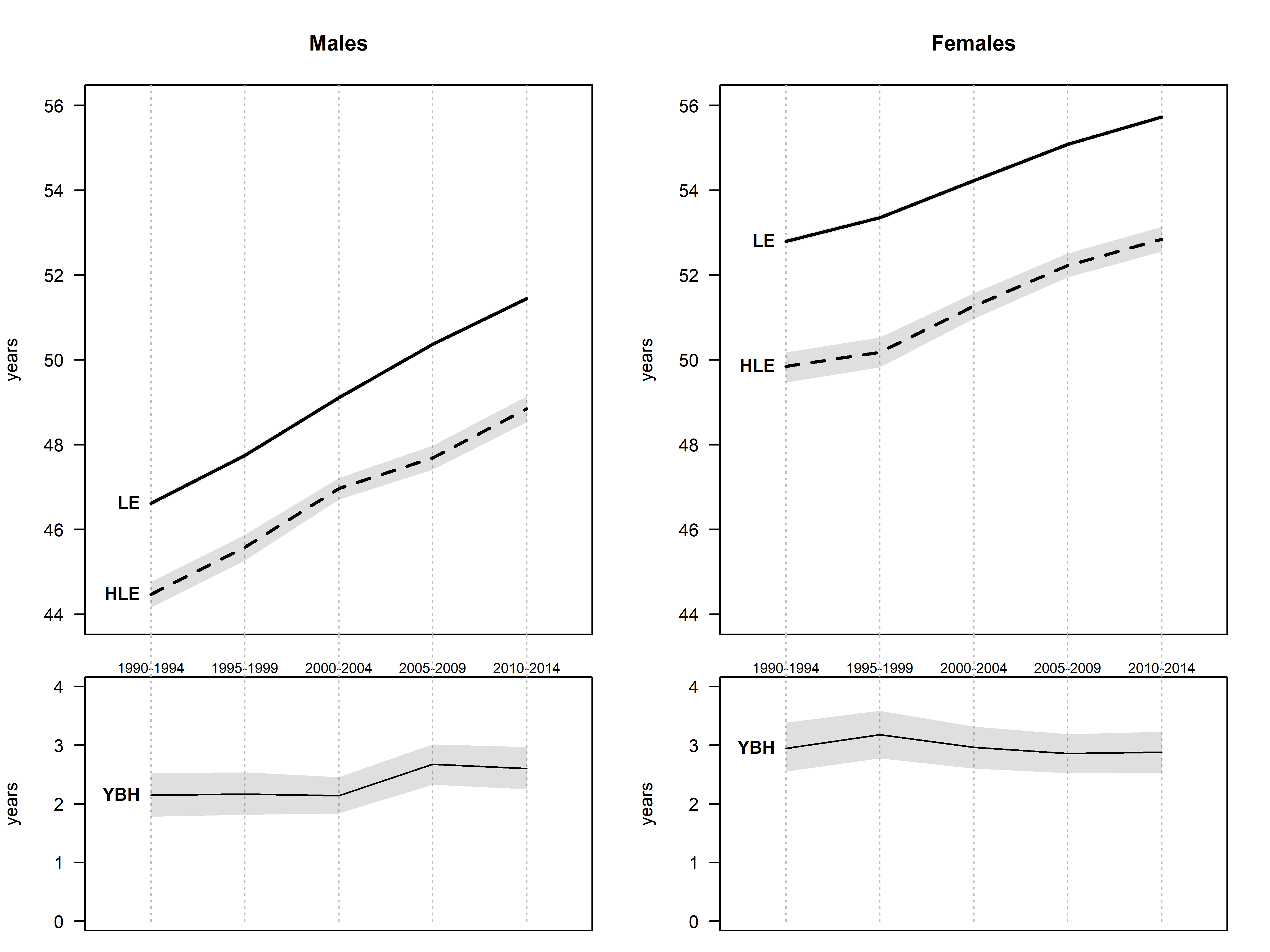


Figure 3: Life expectancy (LE), Healthy life expectancy (HLE), and Years of bad health (YBH) among people present at the 1990 census (Swiss National Cohort and Swiss Health Interview Survey, Switzerland, 1990-2014)


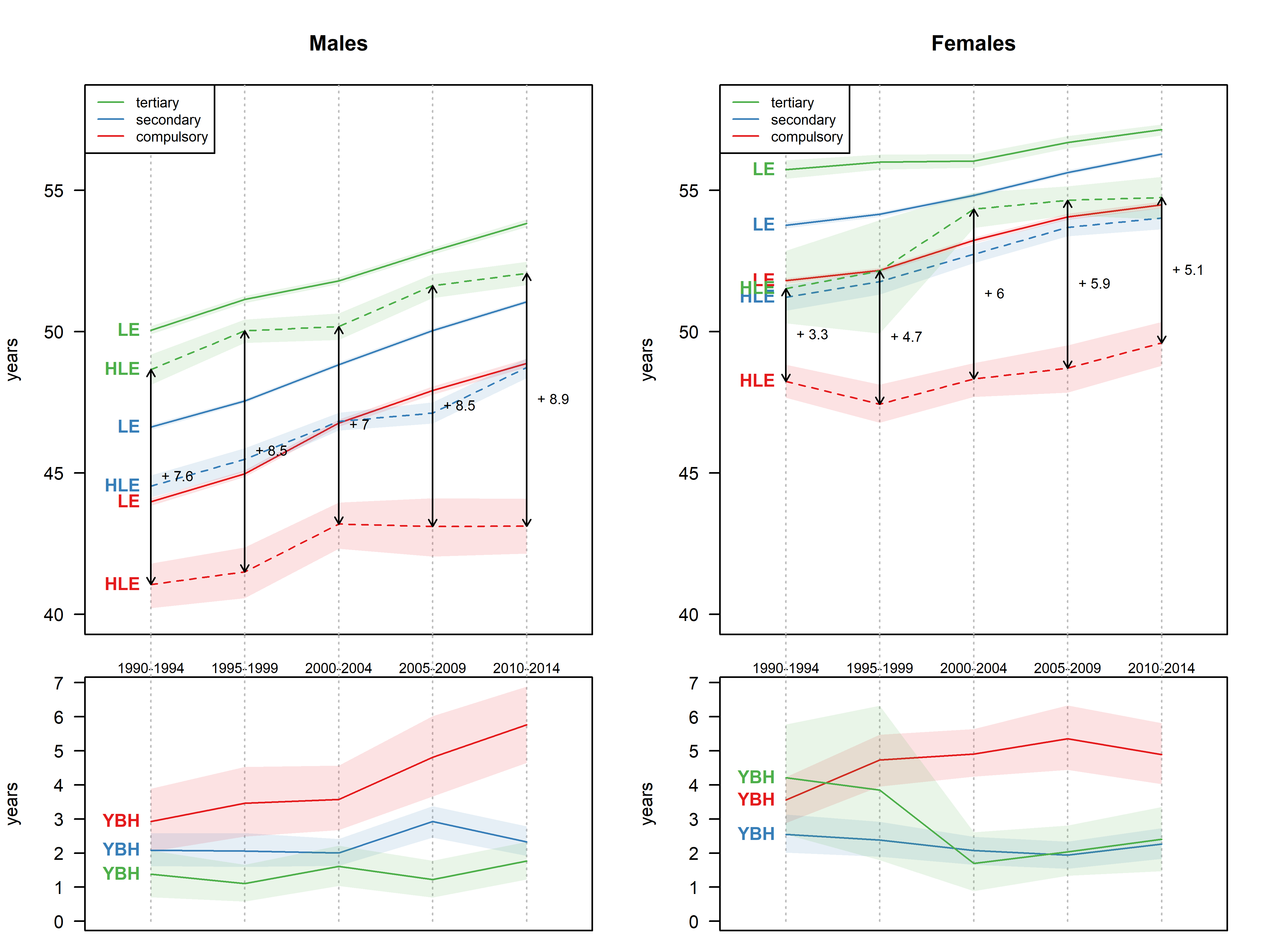


Figure 4: Life expectancy (LE), Healthy life expectancy (HLE), and Years of bad health (YBH) by education level among people present at the 1990 census (Swiss National Cohort and Swiss Health Interview Survey, Switzerland, 1990-2014)

# Online resource 3: age-specific contributions to the changes in YBH

Figure 6: Age-specific contributions to the change in years of bad health by sex (Swiss National Cohort and Swiss Health Interview Survey, Switzerland, 1990-2014)


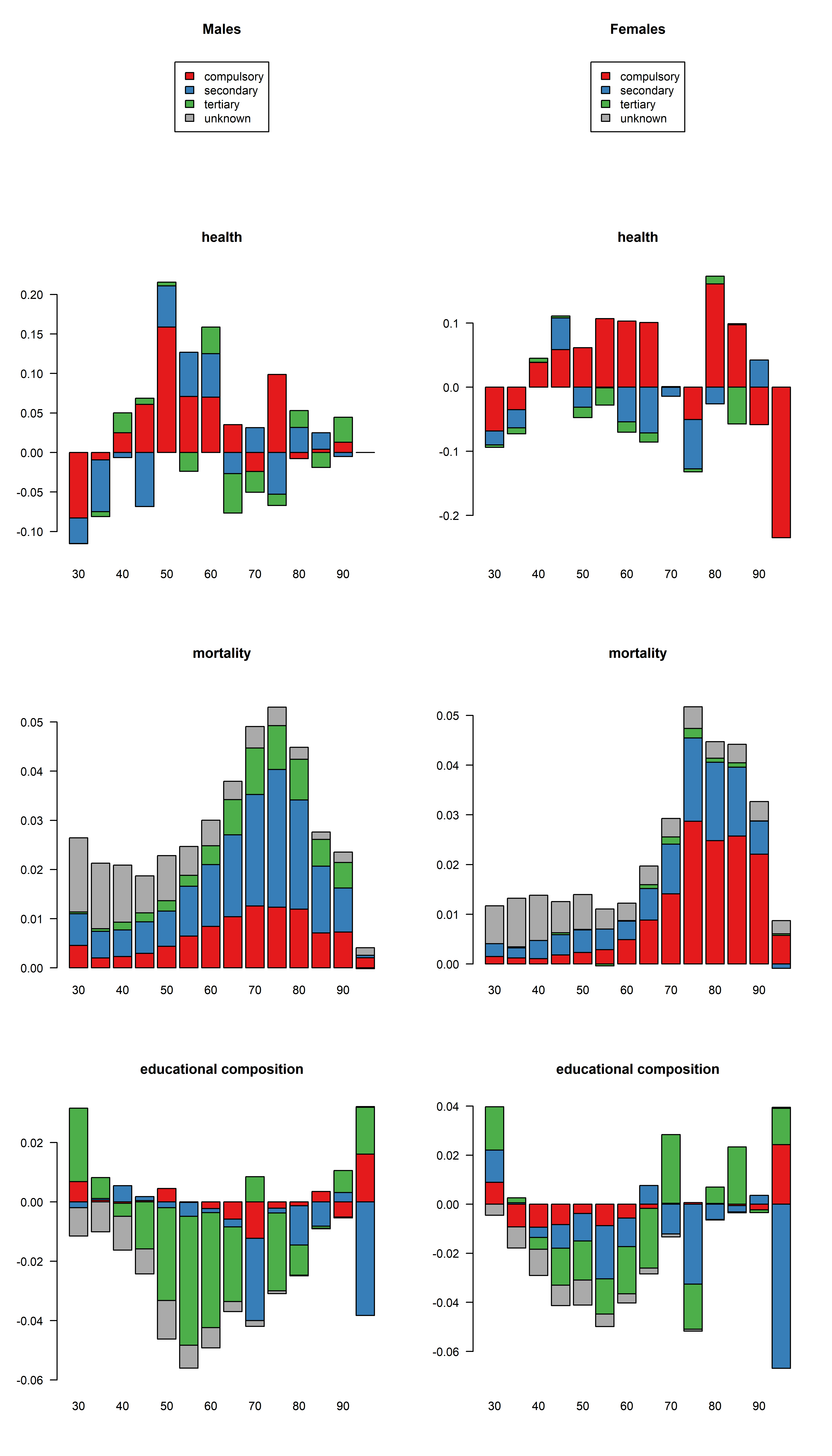


# References

1. Zufferey J. Investigating the migrant mortality advantage at the intersections of social stratification in Switzerland: The role of vulnerability. Demographic Research. 2016;34:899-926.

2. Wanner P, Zufferey J, Fioretta J. The impact of migratory flows on the Swiss labour market. A comparison between in-and outflows. Migration Letters. 2016;13(3):411.
